# Thirty-five years of floristic collections in southern Tuscany (Italy)

**DOI:** 10.3897/BDJ.13.e160516

**Published:** 2025-07-28

**Authors:** Federico Selvi, Stefano Martellos, Matteo Conti

**Affiliations:** 1 University of Florence, Florence, Italy University of Florence Florence Italy; 2 University of Trieste, Trieste, Italy University of Trieste Trieste Italy

**Keywords:** flora of Maremma, georeferenced dataset, herbarium specimens, vascular plants

## Abstract

**Background:**

Floristic knowledge and georeferenced information about vascular plant species distribution in southern Tuscany (Italy) are still poor for supporting effective biodiversity conservation efforts.

**New information:**

A dataset of georeferenced floristic collections from Southern Tuscany, which was developed by the first author between 1989 and 2024, is provided and briefly commented on. The dataset includes data for 4535 herbarium specimens, mostly unpublished, currently preserved in the Herbarium Centrale Italicum at the Natural History Museum of Florence (FI). The specimens belong to 1766 species and subspecies in 122 families of vascular plants. Each record is associated with a Unique Identifier (UID) and information on the collection locality, date, collector(s), and geographical coordinates (WGS84 geodetic datum). Many specimens were collected in areas that were poorly investigated, documenting new sites for several uncommon or phytogeographically relevant taxa. The dataset includes two specimens of *Euphorbiameuselii* Geltman, a forest herb endemic to Southern Italy and new to the flora of Tuscany. Overall, this dataset allows a relevant advancement in the floristic knowledge of central Italy.

## Introduction

Biodiversity conservation, as well as the understanding of species distribution patterns, depends on the amount and quality of the information about species occurrences at the different spatial and temporal scales. For plants, floristic records and associated geographic data are essential elements for in-situ species protection (Wagensommer 2023). Recently, Antonelli et al. (2023) stressed that plant distribution knowledge is crucial to meet the objectives of the Kunming-Montreal Global Biodiversity Framework and the Framework targets for ecological restoration, while Eckert et al. (2024) highlighted that herbarium collections contain a wealth of essential data for conservation biology and remain essential in the age of community science (Greve et al. 2016).

In Italy, the level of information about the distribution of vascular plants is uneven, and several “dark spots” still exist. In these areas, occurrence records are relatively poor and uneven. In Tuscany, botanical exploration started in the Renaissance and led to the relatively large amount of floristic data that is available at present. However, the floristic exploration of the region has remained strongly uneven until recent years (Angiolini et al. 2005), and still today occurrence records from large areas of southern Tuscany are quite scarce. Even in the rich and continuously updated website WikiPlantBase Toscana (Bedini et al. 2015), the density of floristic records from this part of the region is significantly lower than in central and northern parts of Tuscany. This is mostly due to historical and “environmental” factors, including the distance from the main research centres in the region. In the past and until the Second World War, the Tuscan Maremma was difficult to reach, and it was considered a “wild land” with a very low population density, posing threats of various types to people travelling there. As a result, many areas and natural ecosystems have been less impacted than in other parts of the region, thus favoring the conservation of the native flora (Selvi 2010).

Thus, the botanical exploration of southern Tuscany essentially started only in the late 19^th^ century with Stephen Sommier, who collected hundreds of specimens, which are preserved in FI. After him, Adriano Fiori was also active in that territory, making additional collections also preserved in FI and FIAF. Since then, several local floristic checklists of nature reserves and Natura2000 sites were published, mostly between 1990 and 2010 (Frignani et al. 2004, Frignani et al. 2008, Lastrucci et al. 2025). However, most parts of the Tuscan Maremma still retain a rural character with a widespread naturalness even outside protected areas, hosting a rich, albeit understudied, flora.

Until 2010, our collections from these territories were the basis for the compilation of the first critical checklist of the flora of the Grosseto province, including 1925 specific and subspecific taxa (Selvi 2010). In this checklist, however, no geographical references were given for the specimens, and the herbarium collection number was mentioned for only a few taxa of phytogeographical interest, critical taxonomic interpretation, or difficult identification. Hence, the bulk of the information associated with the specimens remained unavailable. The continuous effort in the floristic investigation of this territory led (to date) to the collection of 4535 herbarium specimens that are currently preserved in the Herbarium Centrale Italicum of the Natural History Museum in Florence, Italy (FI H.C.I.).

## General description

### Purpose

The dataset presented in this paper is intended to advance the floristic knowledge of a biodiversity-rich territory of central Italy and support the conservation of its flora. It provides geographical details for 4535 georeferenced specimens, with updated nomenclature and a short textual description of the collection localities. Novel localities for several taxa of phytogeographical and conservation relevance are documented, including specimens of *Euphorbiameuselii*, a forest herb endemic to southern Italy and previously unknown from Tuscany.

## Sampling methods

### Sampling description

About 95% of the specimens in the dataset were collected by the first author; other collectors were S. Sforzi (138), P. Senesi (43), D. Viciani (30), M. Bigazzi, F. Frignani (7) and P. V. Arrigoni (1).

**Figure 1.**
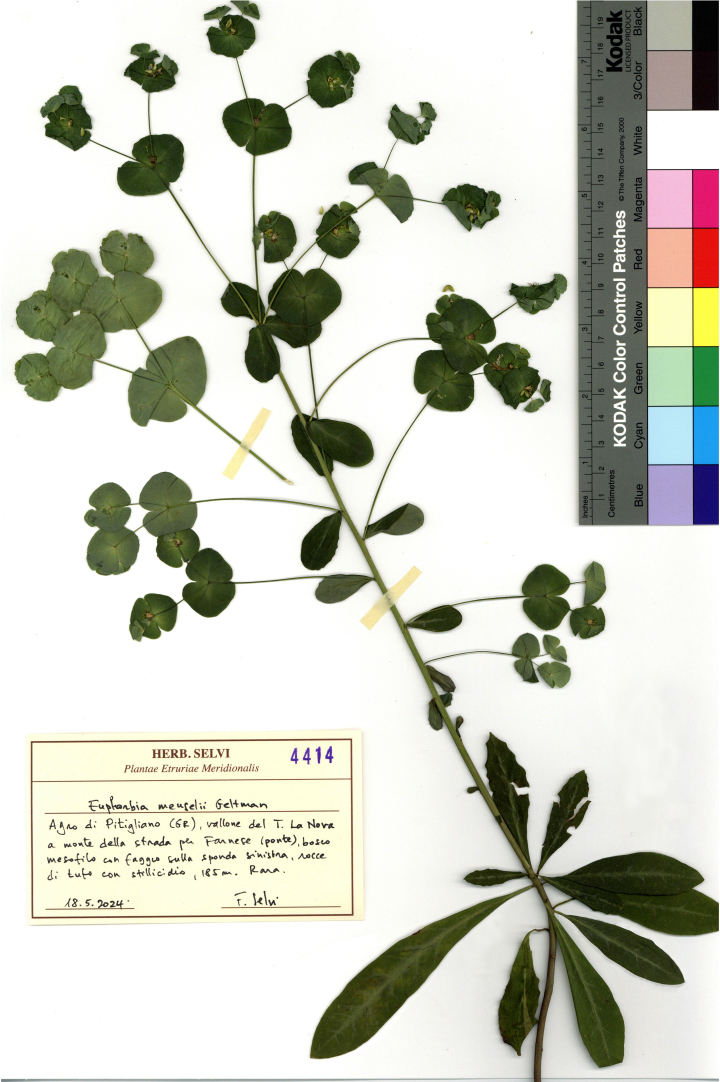
Specimen of *E.meuselii* collected at Fosso La Nova, Pitigliano (no. 4414).

Specimen identification was carried out by the first author on the basis of a wide range of literature sources, among which are Flora d’Italia (first edition, Pignatti 1982; second edition, Pignatti 2017) and Flora Europaea (Tutin et al. 1964, Tutin et al. 1993). The recent Flora Analitica della Toscana by Arrigoni (2016) was also used. In many cases, a comparative study of herbarium material in FI and FIAF was also carried out. Geographical details of gathering localities and a brief textual description of the habitat were normally provided on the specimens’ label, together with soil type and altitude. Toponyms generally followed the 1 : 25,000 maps by the Italian “Istituto Geografico Militare.” However, sometimes unofficial, vernacular locality names commonly used by local people were reported in the label. The data were handwritten in Italian by the author on printed labels created *ad hoc* for this collection series (Herb. Selvi, Plantae Etruriae Meridionalis). The labels’ format has been slightly changed over the years. An example of a label of a recent specimen is shown in Fig. 1.

The specimens were preserved in the author’s private herbarium (Herb. F. Selvi) until 2022, when the whole collection was transferred to the Herbarium Centrale Italicum (FI-HCI), where it is currently preserved (Lastrucci et al. 2023). Before 2021, many duplicates of specimens of phytogeographically relevant species, including those published as floristic notes, were also deposited in FI.

Digital scans or photographs of each specimen were taken and stored during the last ten years; the images are available upon request. Since a national project for the massive digitization of the FI Herbarium is currently going on, high-resolution scans of all the specimens listed in the dataset will soon be available online.

Data of all the specimens were originally entered in an Excel spreadsheet. They include family, taxon name (species and subspecies), gathering locality, date, collector, and ID (collection number). The data were then converted to the Darwin Core format (Wieczorek et al. 2012) to ensure standardization and interoperability with other biodiversity databases. The verbatim name of the first identification is reported if it differs from the currently accepted name. In the period 1988–2005, nomenclature followed Flora d’Italia (Pignatti 1982) and Flora Europaea (Tutin et al. 1964, Tutin et al. 1993), while in the period 2006–2017, it followed Conti et al. (2015). For the purposes of this work, names were revised according to the recently published “A second update to the checklist of the vascular flora native to Italy” (Bartolucci et al. 2024) and “A second update to the checklist of the vascular flora alien to Italy” (Galasso et al. 2024); these names are also adopted in the Portal to the Flora of Italy (Martellos et al. 2020).

The transcription of the gathering sites was not always made verbatim but was sometimes shortened. Georeferencing of the localities for the specimens gathered before 2022 was done a posteriori between 2023 and 2024 on the basis of information reported on the label and, above all, on the first author’s personal memory of the sites visited during 35 years of fieldwork. To this purpose, field notes taken at the time were also used. For each gathering site, geographical coordinates based on the WGS84 system are provided with a level of accuracy of 5 km before 2022 and < 1 km for the collections from 2022 to 2024. Gathering sites were mapped using QGIS v. 3.22. (QGIS 2025)

## Geographic coverage

### Description

**Figure 2.**
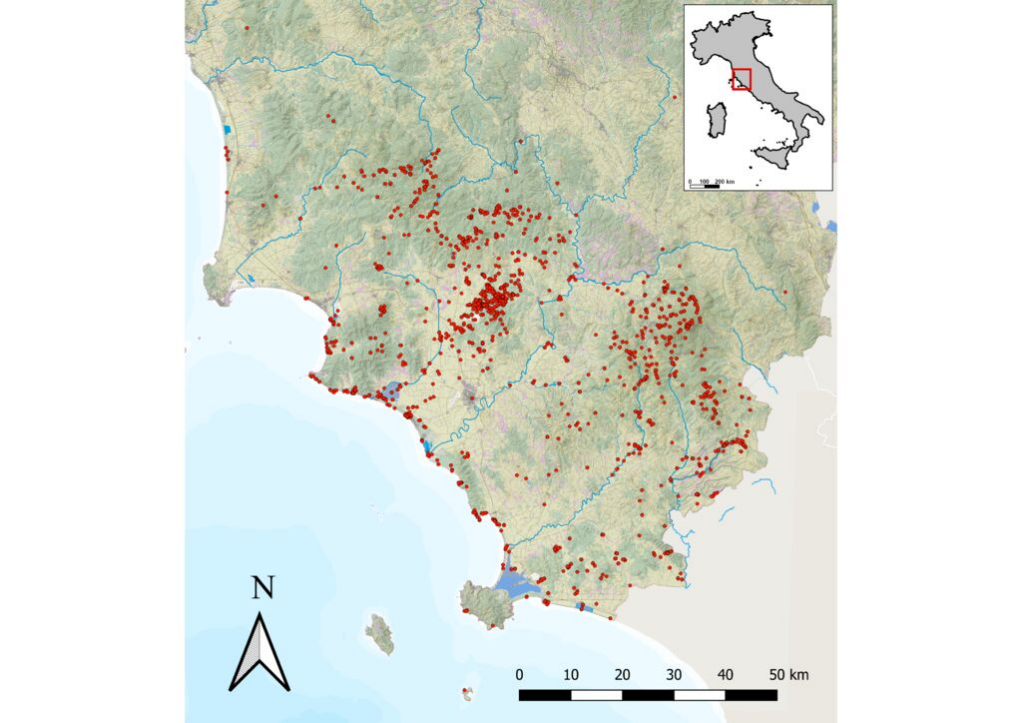
Map of the floristic collection sites.

Floristic investigation was mainly focused on the province of Grosseto, the southernmost and largest one in Tuscany (4503.12 km²; the 16^th^ largest in Italy). The main environmental and vegetation features of this territory, along with phytogeographical subdivisions, are outlined in Selvi (2010) and in Selvi in Giovacchini et al. (2015). A small number of specimens of phytogeographical relevance were also collected in the southern parts of the provinces of Siena, Pisa, and Livorno, south of the river Cecina, which represents the northern limit of the historical region of the Tuscan Maremma. A map of the collection sites in Tuscany is shown in Fig. 2. A specimen from the province of Viterbo (Latium) is also included among the collections (not shown in Fig. 2).

Median Latitude: 42.85712; Median Longitude: 11.23756.

### Coordinates

South-bound Latitude: 42.25720 and North-bound Latitude: 43.39770 Latitude; West-bound Longitude: 10.53680 and East-bound Longitude: 12.13690 Longitude.

## Taxonomic coverage

### Description

**Figure 3.**
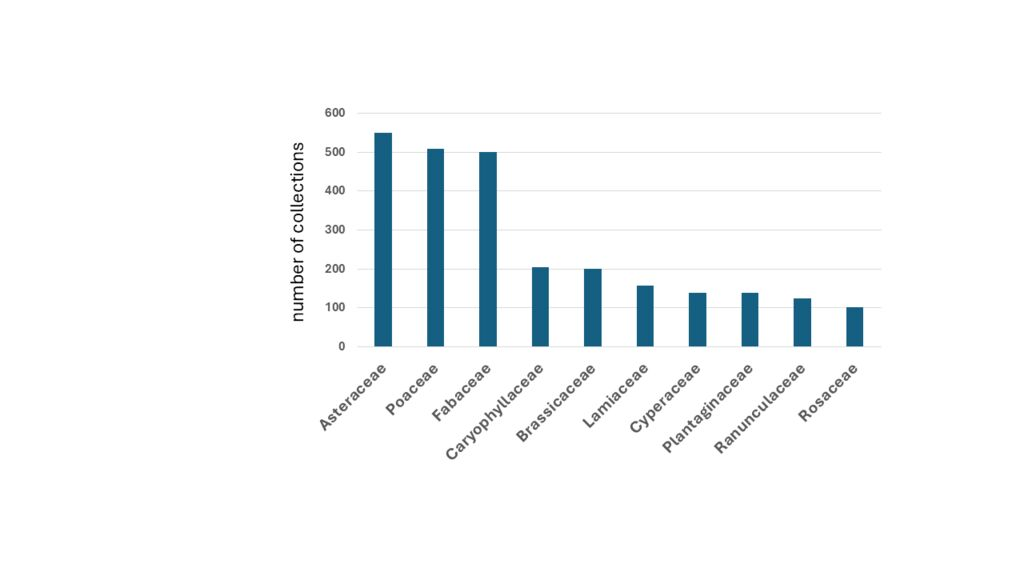
Most represented families.

The specimens belong to 1766 species and subspecies in 122 families of vascular plants. Asteraceae (550), Poaceae (509), and Fabaceae (500) are the most represented; ten families are represented by more than one hundred specimens (Fig. 3). Family Orchidaceae is underrepresented, since specimen gathering was often avoided for conservation purposes. The specimens include two hybrids, *Acer* x *martini* Jord. and *Quercus* x *morisii* Borzì. Overall, the dataset covers c. 90% of the vascular flora of the Tuscan Maremma (based on Selvi 2010), nearly half the native flora of Tuscany (c. 47.9%), and 20.5% of the Italian flora, according to Bartolucci et al. (2024). According to Galasso et al. (2024) and Bartolucci et al. (2024), the specimens of alien taxa are c. 3.7%, including those that are alien at the regional level, such as *Genistaetnensis* Raf. Thirty-eight specimens are invasive neophytes (N INV), 15 are naturalized neophytes (N NAT), 4 are invasive archaeophytes (A INV), and 5 are naturalized archaeophytes (A NAT). Two specimens are cryptogenic, according to Galasso et al. (2024). Among the invasive neophytes, there is *Oenotherastucchii* Soldano (new to the flora of south Tuscany), which was collected in the territory of Civitella Marittima (UID: 4526). The total number of taxa that are not native to Tuscany is 48, i.e., 2.7% of the flora in the dataset and c. 7% of the alien flora of Tuscany inventoried to date.

**Figure 4.**
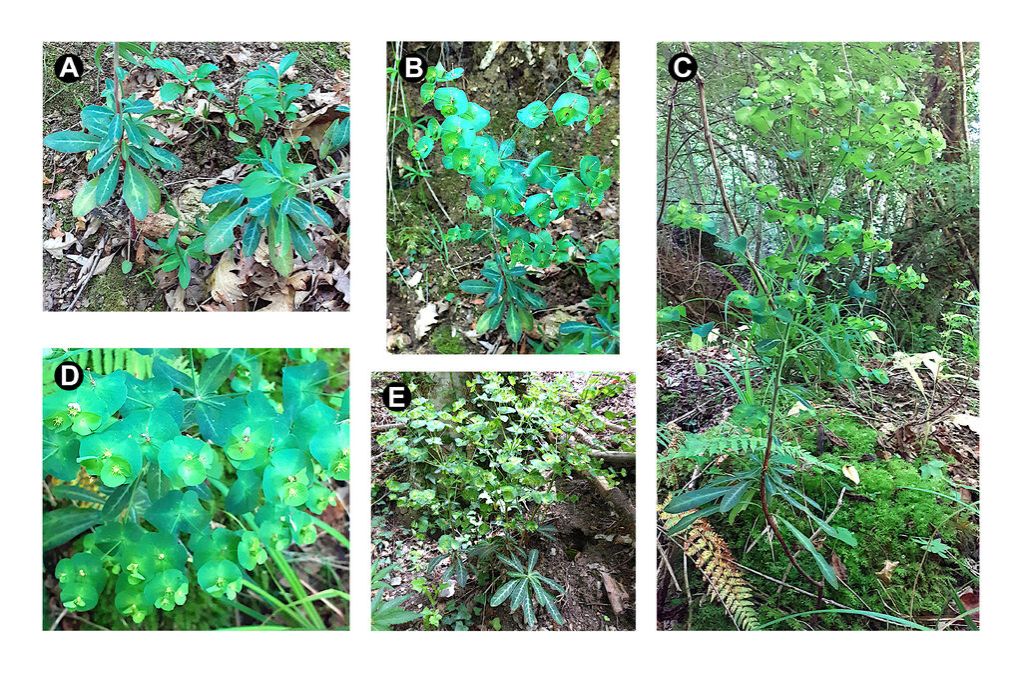
Field photographs of *E.meuselii* Geltman from south Tuscany (A-D) and Calabria, Mt. Pollino area (E).

Among the most relevant specimens in the dataset, there is *Euphorbiameuselii* Geltman, a forest herb currently known only from southern Italy and thus new to the flora of Tuscany. The present report is the first one for this region, and is therefore briefly commented. This species was collected in two sites of the valley of the river Fiora, at the southernmost part of the province of Grosseto, in the territory of Pitigliano, in 2023 (UID: 4333) and 2024 (UIDs: 4414 and 4415; Fig. 3). The first site was along the stream Fosso La Nova, a left tributary of the Fiora River, which flows in a deep gorge carved into effusive volcanic rocks of ignimbritic type (“tufo”). Here, a small population was growing close to the stream bank, on its left hydrographic side, facing northeast (Fig. 4C). This population is part of the shaded understorey of a mesophilous forest, which hosts an extra-zonal population of *Fagussylvatica* L. (UID: 4335) rooted on a steep escarpment with dripping water from uphill sources. The second specimen (UID: 4415) was gathered along the river Fiora, where it was growing in the understorey of a mixed mesophilous forest on the right bank of the river, with a north-facing slope aspect (Fig. 4A-B). The plants of *E.meuselii* from both these sites were in the late-flowering to early-fruiting stage and showed the diagnostic characters that distinguish this species from its widespread relative *E.amygdaloides* L. These features are the sparsely pubescent to nearly glabrous stems and leaves (rather than usually distinctly short pubescent in *E.amygdaloides*), the evergreen (or almost so) and somewhat coriaceous leaves (rather than herbaceous and deciduous in *E.amygdaloides*), which are dark green and shiny in color, with whitish strips on the adaxial surface along the veins (Fig. 4A-C). The flowering stems bore a lax inflorescence with up to 10 axillary rays, often dichotomous, below the apical umbel (Figs 1, 4C); the 5–6 rays of the umbel were subtended by more rounded leaves than usual in *E.amygdaloides*, and the bracts of the cup-shaped involucre of the cyathia were also distinctly rounded; the horns of the floral glands were thin and elongated, greenish in color (Fig. 4D). The seeds were ovoid, 2 × 1.6 mm, shiny and black, with an elaiosome allowing for ant dispersal. These features were observed in plants collected by the author in the type locality in the Madonie mountains (Sicily) and in Mt. Pollino (Calabria; Fig. 4E), though the Sicilian plants showed more coriaceous leaves than those from either Calabria or Tuscany. *Euphorbiameuselii* is distributed in Sicily (Madonie mountains, Etna) and Calabria (Sila, Mt. Pollino, Serre Calabre) and is reported also from Sardinia and Basilicata (Pignatti 2017, Bartolucci et al. 2024), though the presence in the latter two regions appears doubtful. Hence, its isolated presence in Tuscany shifts the northern distribution limit of this endemic species to some 400 km with respect to the currently known range limit in southern Italy. Further studies on the complex of *E.amygdaloides* in Italy would be useful to better define the limits of the taxa included in it.

## Temporal coverage

### Notes

**Figure 5.**
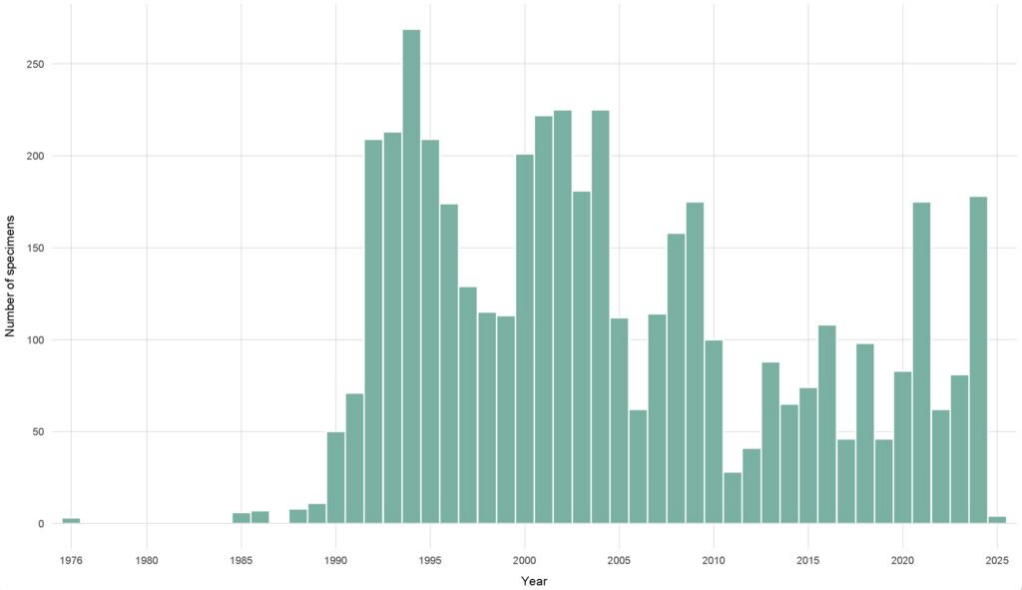
Specimens collected per year.

The earliest specimen was collected in 1976, and all the others between 1985 and 2025 (January 4). The great majority were collected after 1989. The temporal distribution of the collections per year is displayed in Fig. 5.

## Usage licence

### Usage licence

Other

### IP rights notes

Creative Commons Attribution License (CC BY 4.0)

## Data resources

### Data package title

Georeferenced vascular plant collections in south Tuscany (Italy)

### Resource link

https://doi.pangaea.de/10.1594/PANGAEA.975872

### Number of data sets

1

### Data set 1.

#### Data set name

Georeferenced vascular plant collections in south Tuscany (Italy)

#### Data format

Darwin Core

#### Description

The dataset refers to 4535 selected floristic collections, mainly done by the first author in southern Tuscany (Maremma, Italy) during the last 35 years (1989–2024, Herbarium F. Selvi). The collections are largely unpublished and currently kept in the Herbarium Centrale Italicum at the Natural History Museum of Florence University (FI, H.C.I.). The collections belong to 1766 specific and subspecific taxa with updated names, according to the Portal to the Flora of Italy, in 122 families of vascular plants. Each record is associated with textual information on the collection locality, date, collector(s), ID (collection number), and geographical coordinates in the WGS84. The dataset is deposited in the Pangaea repository.

**Data set 1.** 

Column label	Column description
ID	An identifier for the occurrence (corresponds to dwc:occurenceID).
Samp type	The specific nature of the data record (preservedSpecimen for all records, corresponds to dwc:basisOfRecord).
Family	Taxonomic family to which the organism belongs (corresponds to dwc:family).
Scientific name	Scientific name of the organism (corresponds to dwc:scientificName).
Plant locality	Locality where the occurrence was recorded (corresponds to dwc:locality).
Latitude	Latitude of the location in decimal degrees (corresponds to dwc:decimalLatitude).
Longitude	Longitude of the location in decimal degrees (corresponds to dwc:decimalLongitude).
Date/Time	Date on which the occurrence was recorded (corresponds to dwc:eventDate).
Investigator	A list of names of people responsible for recording the original occurrence (corresponds to dwc:recordedBy).
Catalog No	The identifier for the record within the collection (corresponds to dwc:catalogNumber).
Cont	Name of the continent where the occurrence was recorded (corresponds to dwc:continent).
Country	Name of the country where the occurrence was recorded (corresponds to dwc:country).
Code	Standardised code representing the country (corresponds to dwc:countryCode).
Taxon rank	Taxonomic rank of the scientific name (corresponds to dwc:taxonRank).
Language	Language of the resource (corresponds to dwc:language)
